# Supplementary figures and images for: Coordinated neuron-glia regeneration through Notch signaling in planarians
Source: PLoS Genet. 2025 Jan 27;21(1):e1011577. doi: 10.1371/journal.pgen.1011577 (PMC11801701; doi:10.1371/journal.pgen.1011577)

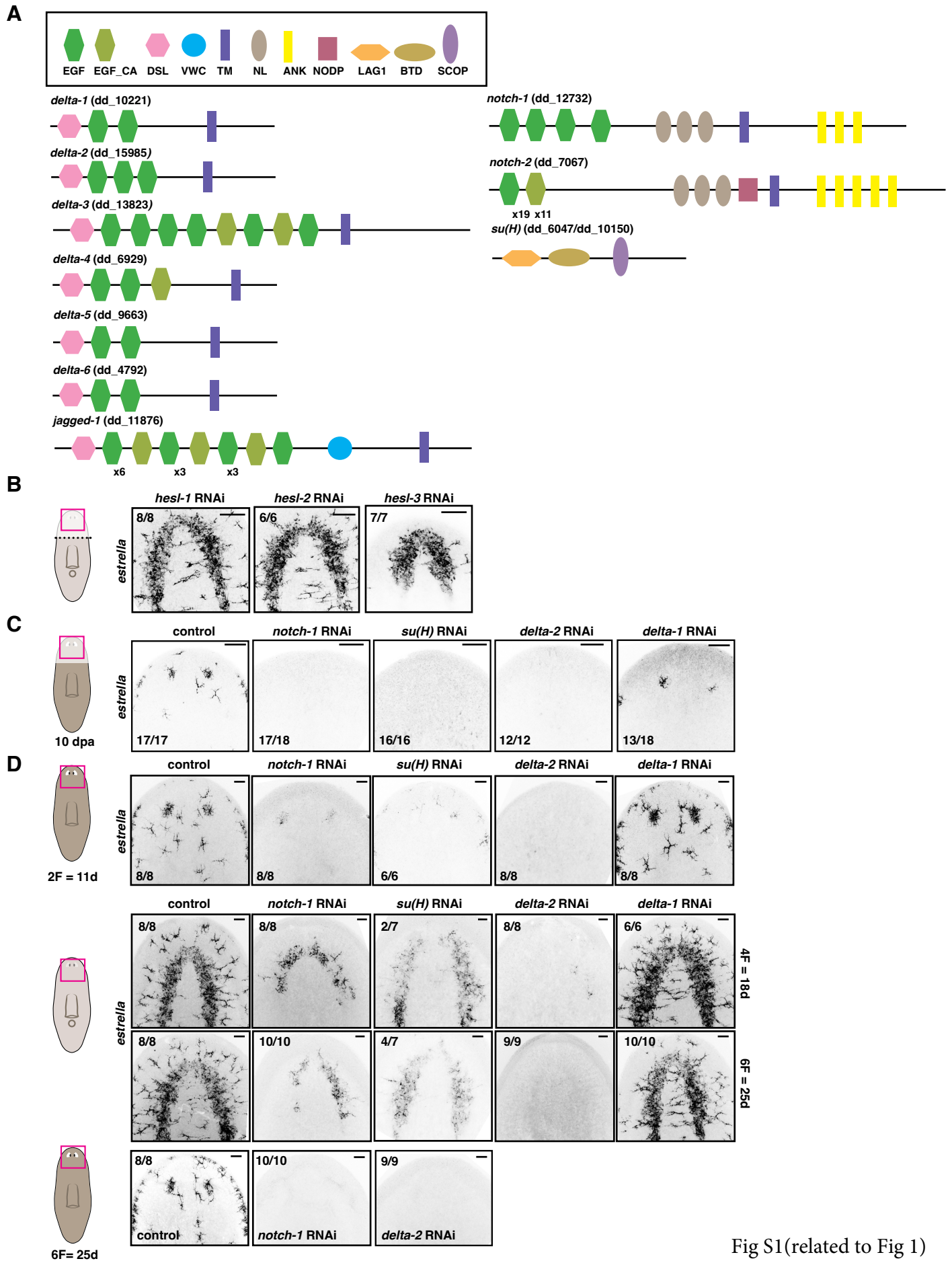

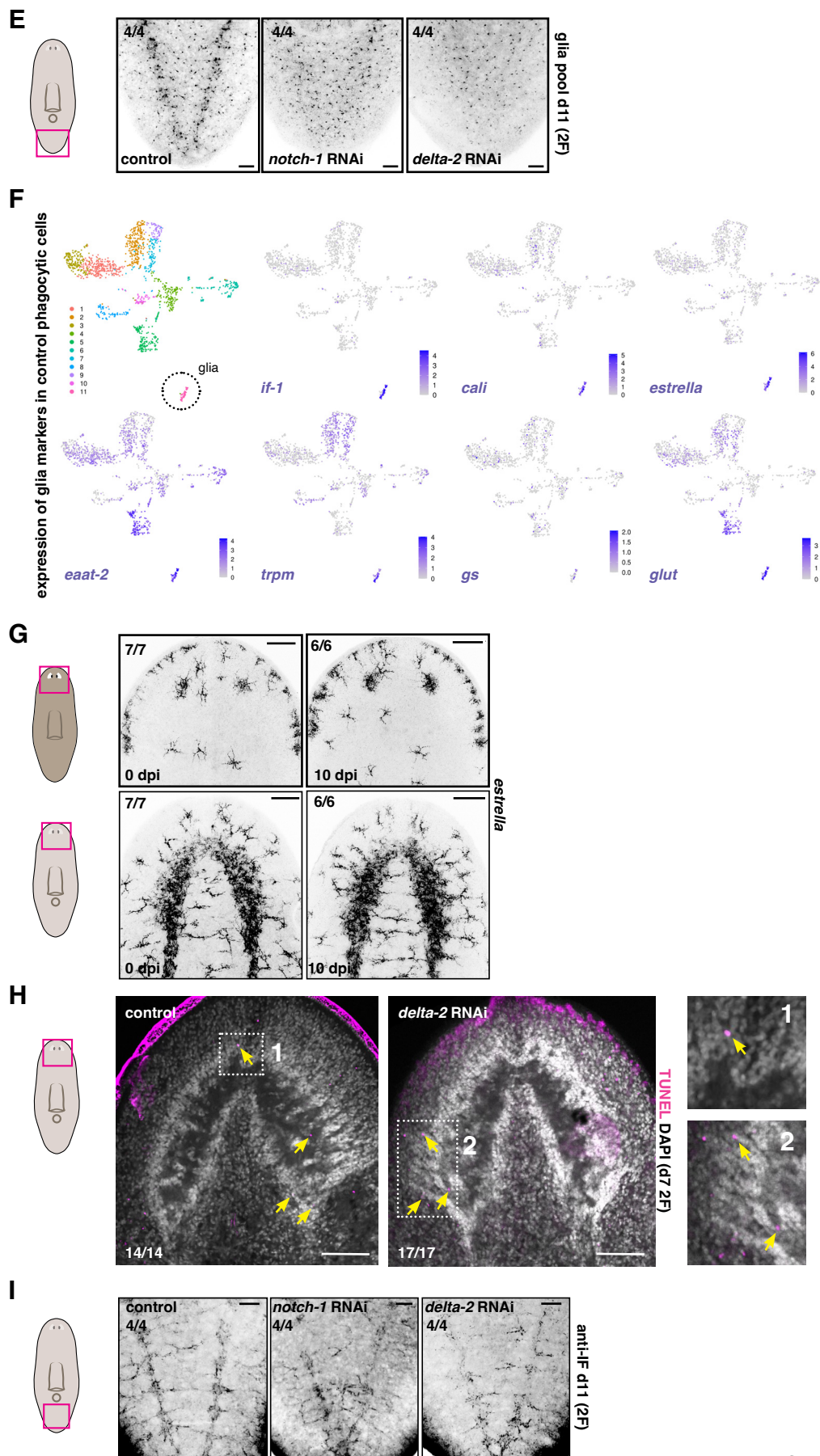

Fig S1 cont'd (related to Fig 1)

Supplement: S1 Fig — (A) Protein domain structure of Notch signaling components used in this study. (B-E) FISH shows glial cell expression during regeneration (B,C) and in uninjured animals (D, E) following different RNAi conditions. (F) UMAP plots show expression of glia markers used in S1E Fig and estrella within the phagocytic cell clusters. (G) estrella+ glia expression in irradiated uninjured animals (dorsal view top, ventral view bottom). (H) TUNEL staining shows a similar number of apoptotic cells within the neuropil. (I) Some IF protein is still present after RNAi treatment. FISH images are representative of one experiment (E,G,I) and at least two independent experiments (B-D, H). Numbers indicate animals displaying the phenotype shown. Cartoons display area imaged, dark animal cartoon indicates dorsal view, light shade cartoon indicates ventral view. Anterior, up. Scale bars, 100 μm. (PDF) [file pgen.1011577.s001.pdf]

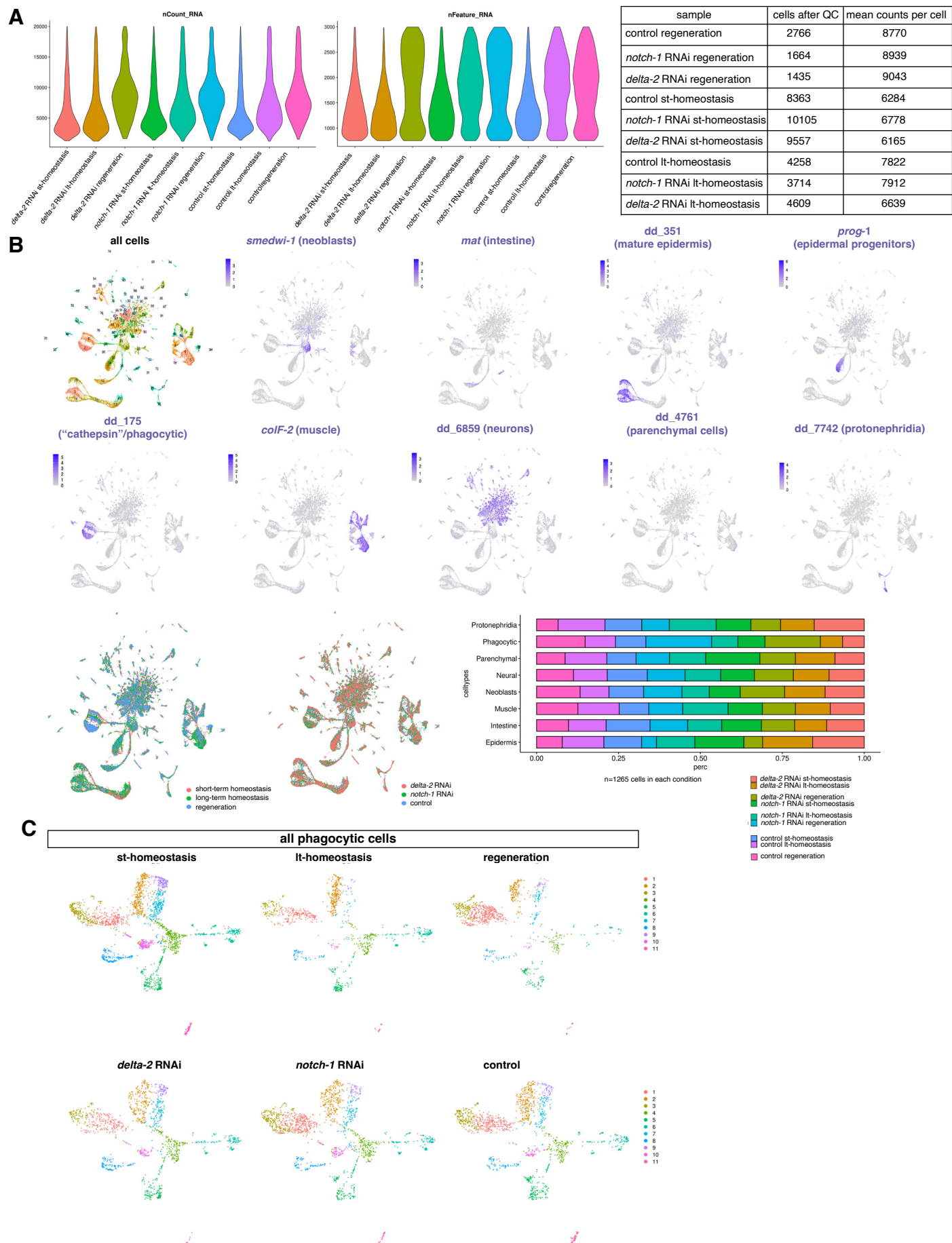

Fig S2 (related to Fig 2)

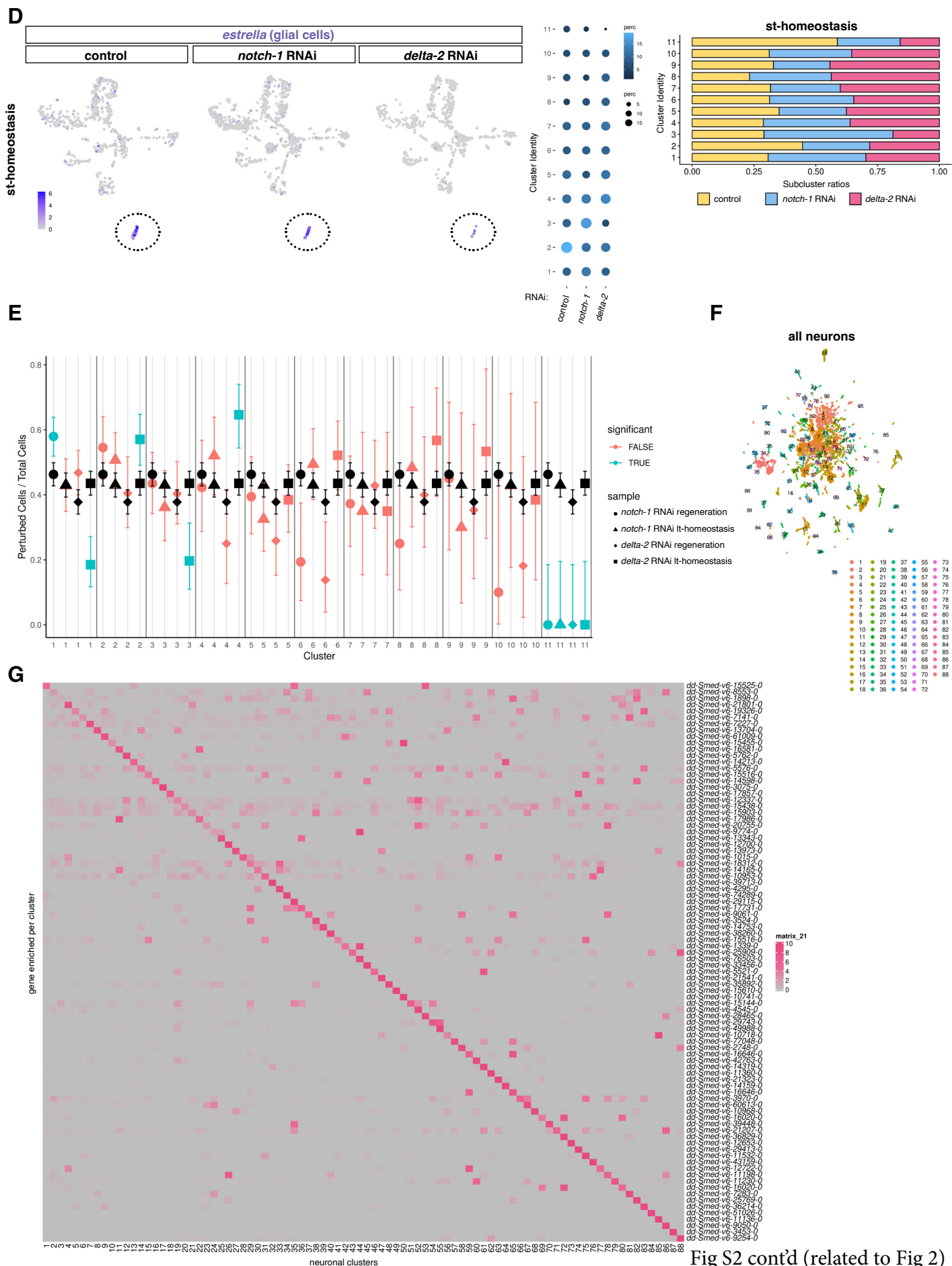

Fig S2 cont'd (related to Fig 2)

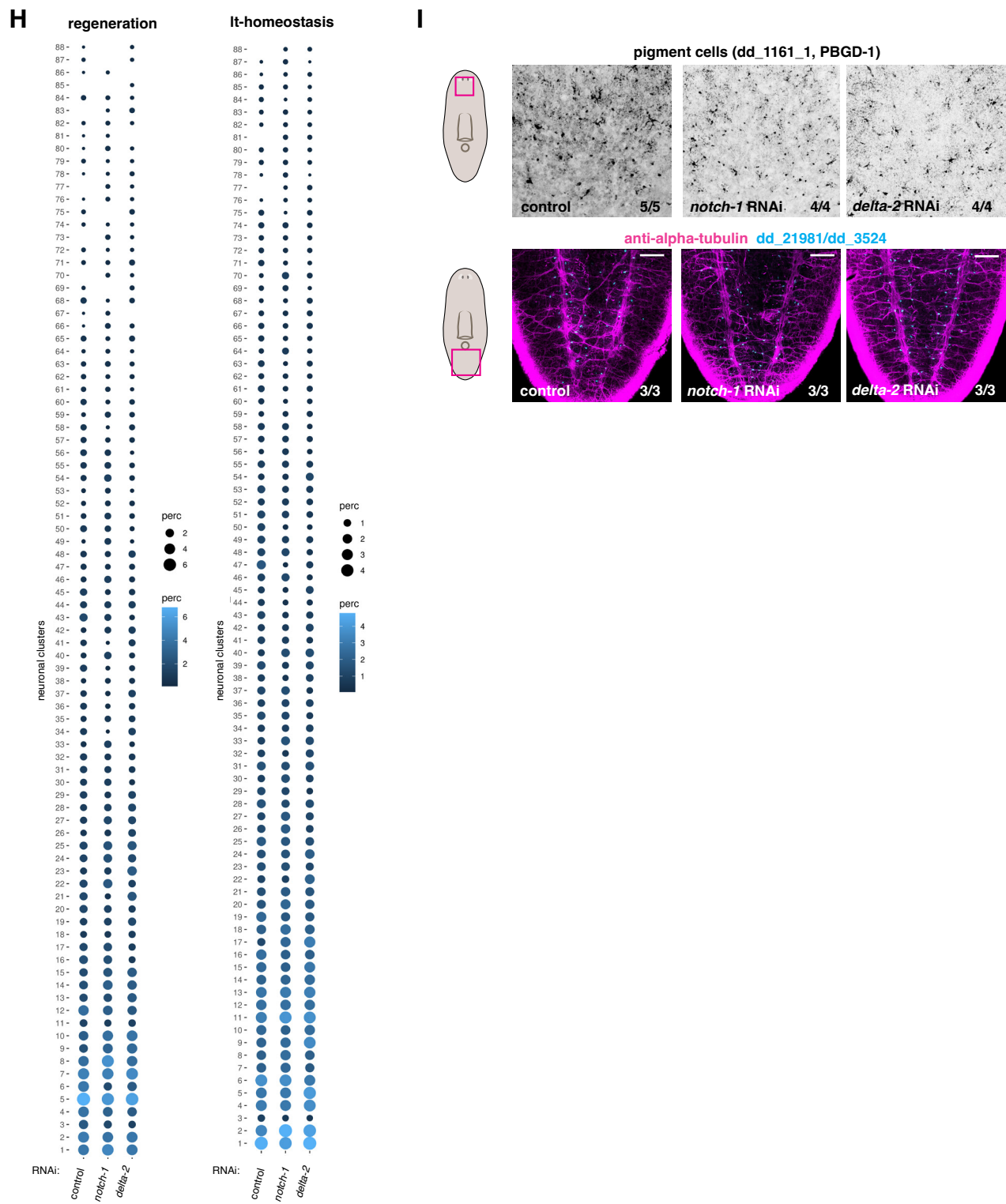

Fig S2 cont'd (related to Fig 2)

Supplement: S2 Fig — (A) QC for all lanes. Violin plots show number of UMIs (left) and genes (center) per cell in each of the sequencing lanes. st, short-term; lt, long-term RNAi treatments. Table (right) shows total number of cells after QC per lane, and mean reads per cell. (B) UMAP plot (top left) shows all cell clusters. UMAP plots show expression of specific tissue markers. UMAP plots show contribution of cells to different clusters per treatment (bottom left) and RNAi condition (bottom center). Graph (bottom right) shows contribution of every lane to each tissue. (C) Split UMAP plots show contribution of cells to different phagocytic clusters per treatment (top) and per RNAi condition (bottom). (D) Split UMAP plots (left) show estrella expression, dot plot (center) shows percentage of each phagocytic cluster, and bar graph (right) shows proportion of each phagocytic cluster in short-term homeostasis per RNAi condition. (E) Dot plot of ratios of perturbed cells to total cells for phagocytic clusters. Black symbols indicate expected ratios from all cells. Colored symbols indicate observed ratios from cells of a given cluster (blue = not significant, red = significant, p < 0.0002). (F) UMAP plot shows neuronal clusters for all lanes. (G) Heatmap shows top specific markers for each neuronal cluster. (H) Dot plots show percentage of each neuronal cluster in regeneration and lt-homeostasis in each RNAi condition. (I) FISH (top) shows no overt difference in pigment cells among different RNAi conditions after long-term RNAi. Immunostaining (bottom) shows normal axonal projections and neuronal numbers in long-term homeostasis RNAi animals. FISH images are representative of one experiment. Numbers indicate animals displaying the phenotype shown. Cartoon displays area imaged. Anterior, up. Light shade cartoon indicates ventral view. Scale bars, 100 μm. (PDF) [file pgen.1011577.s002.pdf]

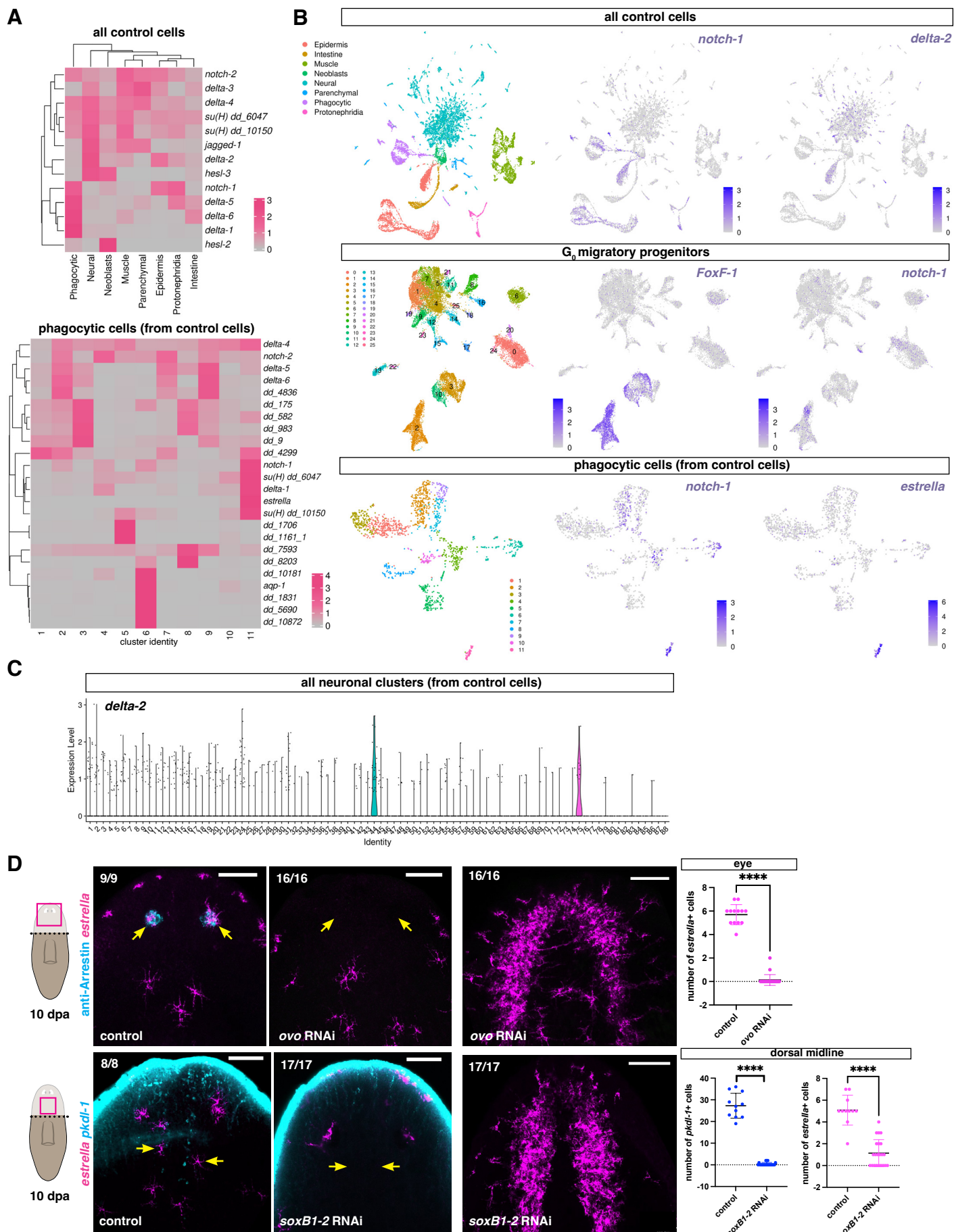

Fig S3 (related to Fig 3)

E

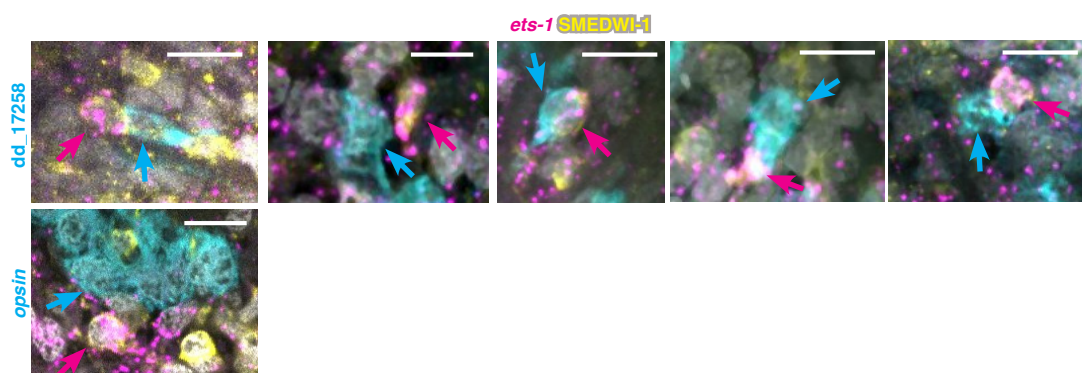

Fig S3 cont'd (related to Fig 3)

Supplement: S3 Fig — (A) Heatmaps show expression of Notch signaling components in different tissues (top) and in phagocytic clusters (bottom). (B) UMAP plots show expression of (top) notch-1 and delta-2 in all cells from control RNAi conditions, (center) FoxF-1 and notch-1 in G0 migratory progenitors [4], and (bottom) notch-1 and estrella in phagocytic clusters. Left UMAP plots in each row show contribution of cells to different respective clusters. (C) Violin plot shows expression of delta-2 in neuronal clusters from control RNAi conditions. (D) Immunostaining and FISH (top row) show expression of estrella+ glial cells (magenta) and photoreceptor neurons (Arrestin+, cyan) in different RNAi conditions. Double FISH (bottom row) show expression of estrella+ glial cells (magenta) and pkdl-1+ neurons (cyan) in different RNAi conditions. Left and center panels, dorsal view; right panels, ventral view. Yellow arrows point to eye glia (top) and dorsal midline glia (bottom). FISH images are representative of two independent experiments. Numbers indicate animals displaying the phenotype shown. Graphs on the right show mean ± SD, analyzed by unpaired Student’s t-test, p-value< 0.0001. (E) FISH and immunostaining shows proximity of ets-1+SMEDWI-1+ cells to delta-2-expressing neurons (opsin+ photoreceptors and dd_17258+ sensory neurons). Cartoons displays area imaged. Anterior, up. Scale bars, 100 μm (D), 10 μm (E). (PDF) [file pgen.1011577.s003.pdf]

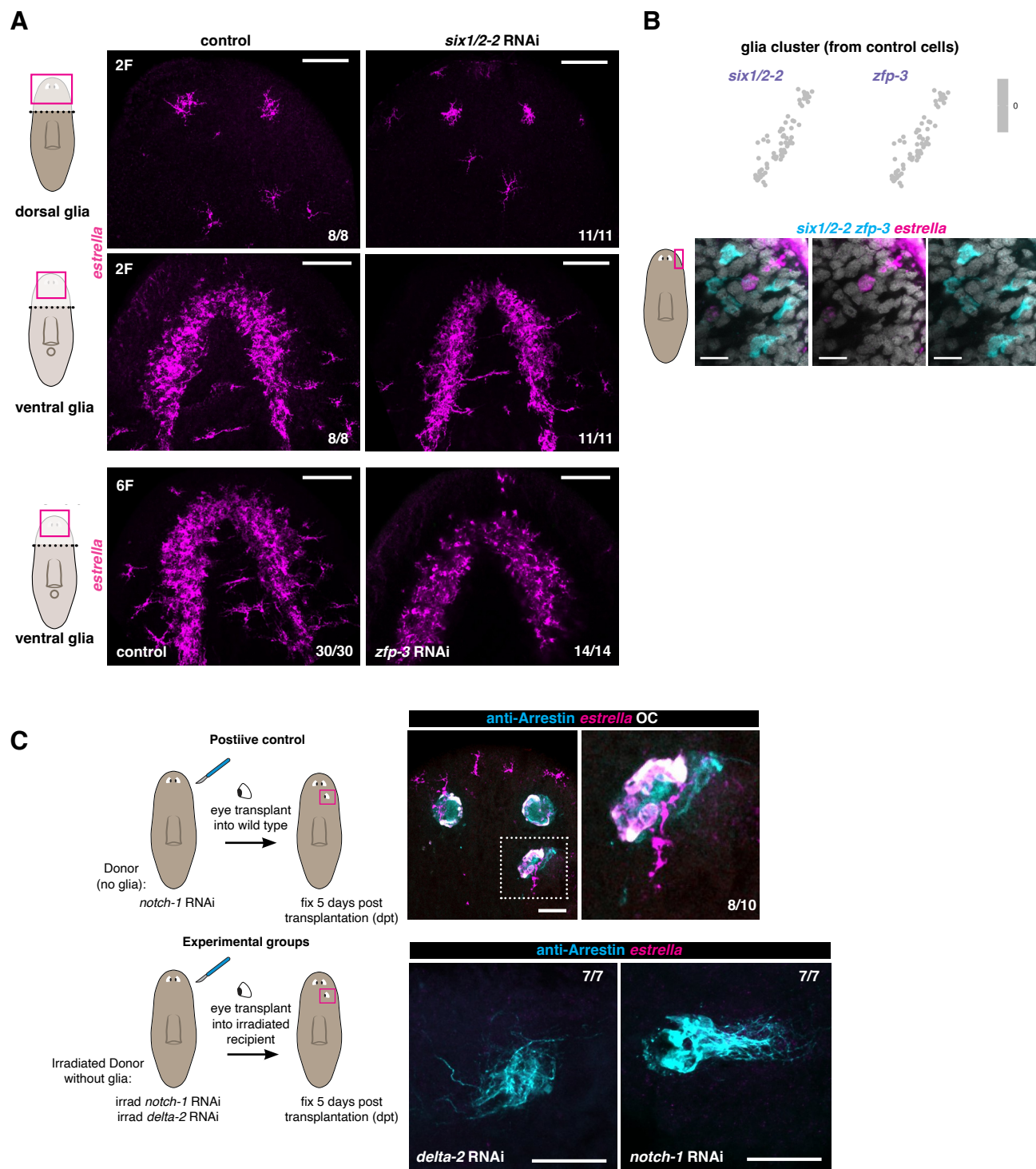

Fig S4 (related to Fig 4)

Supplement: S4 Fig — (A) FISH shows estrella+ glial cells in different locations. Top row, dorsal view. Center and bottom rows, ventral view. FISH images are representative of two independent experiments. Numbers indicate animals displaying the phenotype shown. 2F, two RNAi feedings; 6F, six RNAi feedings. (B) UMAP plots and FISH show lack of expression of the TF six1/2-2 and zfp-3 in the glia cluster (estrella+ cells). (C) Cartoons on the left summarize the experimental setup. FISH shows that glia can differentiate after day 5 post eye transplantation of notch-1 RNAi animals into wildtype recipients. However, no glia differentiation is observed if eye transplant donors and wildtype recipients were lethally irradiated. Cartoons display area imaged, dark animal cartoon indicates dorsal view, light shade cartoon indicates ventral view. Anterior, up. Scale bars, 100 μm (A), 10 μm (B), 50 μm (C). (PDF) [file pgen.1011577.s004.pdf]
