# Supplementary material for: Coordinated neuron-glia regeneration through Notch signaling in planarians
Source: PLoS Genet. 2025 Jan 27;21(1):e1011577. doi: 10.1371/journal.pgen.1011577 (PMC11801701; doi:10.1371/journal.pgen.1011577)
Supplement: Attachment — Submitted filename: reply to reviewers_120524.docx [file pgen.1011577.s011.docx]

Reviewer's Responses to Questions

**Comments to the Authors:
Please note here if the review is uploaded as an attachment.**

Reviewer #1: Functional regeneration requires the restoration of different cell types in the correct place and abundance. In highly-regenerative planarians, neurons and glia are regenerated from distinct progenitors. This study investigates the role of Delta and Notch signaling to specify glia. The authors demonstrate that RNAi knockdown of notch-1 or delta-2 leads to an absence of glia, both after regeneration and in uninjured planaria. They employ single cell RNA sequencing to hypothesize that delta-2 expressed by neurons and notch-1 expressed by glial progenitors is required for glial differentiation. They support this hypothesis by depleting specific neuronal populations and observing a loss of only the associated glia. In addition, through transplantation experiments they demonstrate that delta-2-expressing photoreceptor neurons are sufficient for ectopic glial formation. Taken together, this is a convincing study with broad relevance to the field of regeneration that supports the model that Delta-2+neurons drive glial progenitor differentiation and glial maintenance via Notch-1.

**Thank you to the reviewer for the interest and support of this paper.**

Minor comments
1) While most biologists will be familiar with notch signaling, including a sentence that summarizes the pathway and how Notch and Delta proteins function would provide important context.

**We added a brief summary of the Notch pathway in the introduction section.**

2) The authors should double check their references in the text to figure 2 as they are mislabeled in the text.

**Thank you. We updated the references to the figures in the text.**

3) It is difficult to see the TUNEL+ cells in figure S1G. Could the authors show this channel independently of the DAPI?

**We added a zoom in panel to show the TUNEL+ cells (new Fig S1H).**

4) A lot of the labels on figures related to scRNAseq are too small to be readable. In addition, figure 3C is too small to be interpretable.

**We changed the font size of the labels.**

5) The authors knockdown notch-1, delta-2, or su(H) and observe deficits in negative phototaxis behavior. In the text, they write “to determine whether glia absence affects behavior”, yet it is possible with these manipulations there are deficits independent of glial absence that could be impacting behavior too.

**It is possible; we added a sentence in the text clarifying this point.**

5) The authors should include labels in Fig 3E for the colors of the image.

**We added the labels to the image.**

5) The authors state that the TF-encoding genes six1/2-2 and zfp-3 were not expressed in glial cells, but they cite their scRNAseq data as evidence of this lack of expression. While this approach can argue for expression of a gene in a specific cell-type, the opposite may not be true due to sequencing depth. To make this claim, the authors should do in situs or otherwise validate in an independent way that these transcription factors are not in glia.

**We added a double FISH staining showing that there is no detectable co-expression of these TFs and the glia marker *estrella* (new Fig S4B).**

Reviewer #2: This is a truly remarkable and elegant study showing that planarian neurons induce terminal differentiation of glial progenitors via Delta/Notch signaling. Planarian neurons are known to form prior to glia in regeneration and both cell types differentiate from distinct progenitor cell types, so how the animal can precisely coordinate the production of these closely associated cell types remained unclear. First, the authors show that delta-2 or notch-1 RNAi caused failure of glial differentiation and maintenance of gene expression, identifying new regulators of this cell type. Expression of these factors was selective to several types of neurons (delta-2+) and glia (notch-1+), suggesting a potentially direct interaction mediating the decision to terminally differentiate glia. This included examples of photoreceptor neurons along with associated glial cell types from the planarian eye, which is a tractable and simple organ system. Eyes from either notch-1 or delta-2 RNAi animals lacked associated glia, while transplantation of eyes from notch-1(RNAi) animals into wild-type animals, but not from delta-2(RNAi) animals into wild-type animals, enabled the formation of glia onto the transplanted photoreceptor neurons. Therefore, the authors' experiments nicely argue that delta-2+ neurons instructively, and likely directly, commit the differentiation of eye-associated notch-1+ glial cells. Along the way, the authors develop an entirely new sensitized behavioral assay to show that glia are essential for planarians to robustly avoid light after experiencing a sensory overstimulation. They also find, intriguingly, that the planarian nervous system exhibits a darkening of tissue when glia are lost, a mysterious phenomenon not yet fully explained but present across species, suggesting planarians could also be used to understand the function and pathology of this condition. Together, their findings reveal a mechanism that enables the coordinated differentiation of associated cell types from distinct progenitors so that final organs attain appropriate numbers of each cell type.

This paper was a joy to read, an “instant classic.” The experiments are beautifully executed, rigorously supportive, and introduce several new methods of analysis in pursuit of the question at hand. The message is broad and important. I don’t believe there are any significant experiments further needed to support the conclusions of this study.

**Thank you to the reviewer for the interest and support of this paper.**

Minor comments:
Suggest adding information to the cartoon in S1F indicating irradiation and dorsal versus ventral so that the figure is more clearly understood without the legend.

**We added a cartoon in Fig 1A to clarify the cartoon images for the rest of the paper.**

Authors’ discretion- For the Fig 2G data, I personally find it easier to read data left to right when controls are indicated first on the left followed by experimentals on the right for each set of treatments.

**We updated all the dot plots with the control to the left.**

Fig 2G it wasn’t clear to me how the statistical test was done or which test was done. I assume authors used t-tests between t=0 and each timepoint within each condition?

**There is no graph in 2G (darkening pictures). We are assuming this note is referring to the behavioral assay. The statistical test used is as the reviewer mentioned, one sample t-test with a Bonferroni correction (as in Atabay, 2018).**

There is a figure callout issue at Figure 2G bottom of page 11 (also please add page numbers). “However, we observed darkening…” should cite to Fig 2F not Fig 2G. Likewise callouts to Fig 2H should be 2G. Earlier callouts in Fig 2 also need editing for example “The glial cluster (cluster 11), by contrast…” should be just Fig 2D I believe.

**Thank you – we fixed the callouts.**

The single-cell RNAseq analysis uses a novel method to disaggregate cells that I believe has not appeared previously in the literature (rather than only CMFB+Collagenase dissociation followed by FACS). What was the reason behind this choice of method, are glia cells too large to FACS sort or this papain+Neurobasal better at preserving live glial cells, or perhaps some other reason?

**Based on a literature search and our own experience, we found that cells with long projections like neurons and glia dissociate better using the papain protocol than the collagenase one.** **We have not used FACS to isolate the eye cells from isolated eyes.**

For the future, and out of curiosity, I wondered if glial-absent animals might undergo excessive darkening after sensory overstimulation, which might point to activity dependent accumulation of the pigment. Also I wondered whether these pigments may be related to known pigmentation pathways in planarians, potentially those involving serotonergic precursor substrates. Perhaps it would be possible to genetically ablate such pigment and have a restoration of behavioral activity following overstimulation, which would argue that the pigmentation from glial dysfunction can have a pathological role in this (or other) systems.

**We did not observe further darkening soon after sensory overstimulation, but we have not assessed later timepoints; this could be a phenomenon worth more study in the future.**

Reviewer #3: PGENETICS-D-24-00869

In this manuscript, Scimone and colleagues define a molecular role for Notch signaling in the coupled regeneration of neurons and glia in planarians. This paper addresses an exciting topic and provides a first glimpse into the function of Notch signaling during whole-body regeneration in planarians. It is an excellent fit for PLOS Genetics and I have two major and a few minor suggestions for the authors to consider.

Major:
1. The major critique that I have is that while delta-2+ neurons are very often proximal to notch-1+ glia, no evidence has been presented to show that delta-2+ neurons contact or are in proximity to glial progenitors themselves (as shown in the model figure, Fig. 4C). This type of evidence would help confirm that Notch signaling is critical for glial specification from progenitors and not for local terminal differentiation or transdifferentiation of glia from a more general phagocytic cell type.

**A couple of technical features make capturing progenitor touching *delta-2+* neurons challenging. We do not have specific glia progenitor markers besides *ets-1* (a common TF marker expressed in all phagocytic cells and their progenitors). A further complication is that glia cells are not that abundant in the eye (only 2 to 3 glia cells) or at the head rim. Therefore, catching the right time to see a glia progenitor near a delta-2+ neuron is very challenging. Further unknowns are the proximity and the pace of differentiation. In any case, we tried using the *ets-1* TF RNA probe together with the SMEDWI-1 Ab to label phagocytic progenitors and the differentiated markers *opsin* (for photoreceptor neurons) and dd-17258 (for sensory neurons). We did observe occasionally an *ets-1*+ SMEDWI-1+ cell near a *delta*+ neuron (several examples are added to a new Fig S3E).**

a. ISH showing phagocytic progenitors proximal to delta-2+ neurons would markedly strengthen the manuscript and go a long way to support the model presented by the authors.

**(see above)**

b. Eye transplantation from lethally irradiated donors into irradiated hosts could confirm that glial engrafting doesn’t occur through a non-stem cell driven mechanism (like transdifferentiation).

**We have now added this experiment. Eyes from lethally irradiated *notch-1* and *delta-2* RNAi animals were transplanted into lethally irradiated WT recipients (new Fig S4C). To make sure that all phagocytic progenitors were depleted from the animal following irradiation, we waited 5 days post irradiation to do the transplants. 5 days after transplantation (10 after irradiation) animals showed signs of neoblast depletion (head regression and curling). We therefore had to fix the animals at d5 instead of d7 post transplantation (as was done for the ones shown in Fig 4). Nevertheless, no glia cells were observed in the irradiated *notch-1* and *delta-2* RNAi transplants, suggesting that transdifferentiation is not occurring. Moreover, d5 post-transplantation was sufficient to observe glia cells near a wild-type *notch-1* RNAi eye transplant (positive control, Fig S4C)**

c. The expected result if Notch signaling is important for terminal differentiation rather than specification would be cells stalled at an immature state after pathway perturbation. This might be similar to what authors report in Supp. Fig. 1E, where there seem to be a number of remaining estrella- cells marked by the glial pool after notch-1(RNAi) or delta-2(RNAi). What are these cells? What is the % loss of pool+ cells using this marker after RNAi? Where are the remaining cells expressing markers of this pool in the clustering data? Is it possible that another phagocytic cluster actually represents immature glia or something capable of becoming glia?

**For the FISH panel shown in Fig S1E we did not use the *estrella* probe as a glia marker. Instead, we used a pool of markers (described in methods) that included *if-1*, *cali*, *glut*, *eaat-2*, *gs* and *trpm*. Many of these markers (except for *if-1* and *cali* that are restricted to glia) are enriched in glia cells but still expressed in other phagocytic cells (we now include UMAP plots showing this, new Fig S1F). In regeneration, the loss of *estrella* cells is complete (100%) in both *notch-1* and *delta-2* RNAi animals (Fig 1B, 1C, S1C) and in long-term homeostasis the loss of *estrella* cells is complete in *delta-2* RNAi animals and only a few *estrella+* cells remain within the neuropil in the *notch-1* RNAi animals (Fig S1D) but none *estrella+* cells were observed in the head rim or eyes (now shown in new Fig S1D) at this time point. These results were further validated by the 10X sc-RNA seq data.**

2. Sarah Elliot wrote a thesis that included data on components of Notch signaling in planarians which is available online at Open Access Theses and Dissertations. Though this is not peer reviewed, I would encourage the authors to consider mentioning and citing this thesis as a prior characterization of the Notch pathway in the main text. In a related point, because the components of this pathway have not been detailed in a peer reviewed paper, I would encourage the authors to include Supp. 1A and a pathway diagram/drawing in the main figures. Expression patterns of these genes, if available, would also be useful. This will provide a starting point for other planarian researchers wanting to dive into this pathway and would improve the impact of this manuscript. I would also be interested in knowing whether the nomenclature in this manuscript matches the nomenclature of genes in the Elliot thesis or whether the numbering of Notch/Delta homologs might differ. Clarity on this point might be warranted so that readers can integrate this manuscript with prior work.

**We did actually reference Sarah Elliott’s dissertation in our original manuscript in Fig S1 legend when we discussed the different ligands and receptors of the Notch pathway. We now moved the reference to the results when we refer to Fig S1A. Expression patterns of some of these genes can be found in Elliott’s dissertation. We showed here expression of *delta-2* by FISH but were not able to detect expression of *notch-1*. However, in Fig S3A we did show expression of all components of the pathway in the 10X single cell seq dataset. For the most part, the nomenclature matches the one in Elliot’s dissertation. But to prevent confusion, we did add all the dd_ids of each gene (Fig S1A) to relate each of them to the Appendix A in the Elliott’s dissertation.**

Minor:
1. Notch-1 is also expressed in the protonephridia (Fig. 3A). Are glia present in this location as well? If not, how do the authors imagine that glial production near these structures is avoided?

**Our model is that *delta-2*+ neurons guide the specification/differentiation of *notch-1*+ phagocytic progenitors into glia cells. Expression levels of *delta-2* in protonephridia are pretty low, and undetectable by FISH. Thus, expression of *notch-1* in protonephridia would not be predicted to result in glia differentiation (as it is the neurons not glia progenitors that are *delta-2*-positive).**

2. One challenge in interpreting Fig. 2C-E is the lack of information about phagocytic cell clusters. To which clusters do categorized cell types like pigment cells belong? How do these clusters relate to previous published work on cell types in this category? Even sharing markers of each subcluster would be helpful when the cluster is uncharacterized.

**In Fig 2C, we include violin plots with the most common marker of each cluster (based on Fincher et al) to identify each of the phagocytic clusters. For example, estrella (marking glia cells) is clearly expressed in cluster 11, dd_1161_1 (a pigment cell marker) is expressed in cluster 5. In addition, our supplementary table 1 shows all the markers enriched in each subcluster.**

3. A more rigorous quantification of changes in non-glial cathepsin+ cell types (via ISH in Fig. 1C and in Fig. 2D, with statistical analyses) seems advisable. Numbers of cells in subclusters 1, 3, and 6 also seem decreased in delta-2(RNAi) animals, but I can’t tell how meaningful this is without statistical testing.

**We have now added a statistical analysis (binomial test, Fig S2E) to better understand the impact of the RNAi conditions in regeneration and long-term homeostasis on the different phagocytic subclusters. As can be seen in Fig S2E the only cluster that is consistently affected under all conditions is the glia cluster. In regeneration, cluster 1 was increased in *notch-1* RNAi, whereas some changes in clusters 1, 2, 3 and 4 were observed in *delta-2* long term RNAi. Future experiments will be required to fully understand the impact of these changes. However, in all *notch-1* and *delta-2* RNAi conditions tested, the cluster that stands out as the most consistently affected is cluster 11 (glia cluster) further confirming our FISH experiments.**

4. The pigmentation observed after loss of glia is interesting. Were there any changes in numbers of pigmented cells (which also belong to the phagocytic cluster) or any changes in pigment-associated transcripts? I’m inclined to think this is pigment accumulation in neurons, but it would be helpful to mention evidence that suggests against other possibilities.

**We added a FISH panel (Fig S2I) showing no difference in pigment cells in long term RNAi conditions (at the time where darkening around the CNS and ventral cords was evident). Moreover, the statistical analysis that we now added in Fig S2E also showed no significant impact on the pigment cell cluster under notch-1 and delta-2 long-term RNAi conditions (cluster 5), suggesting that, as the reviewer’s intuition also points to, most likely the darkening is in the neurons. This phenomenon has been previously observed in other systems and we discussed potentially explanations in the text.**

5. It would be nice for the authors to comment on why they believe su(H)RNAi animals experience more marked behavioral impacts. The statistical tests used for the behavioral analysis also seem to mark differences for each sample from time zero to each subsequent time point, while a test to analyze differences between RNAi conditions would be more useful.

**There are multiple possibilities here. RNAi could be more effective with su(H) or because su(H) is a downstream component in the Notch pathway it could have a broader effect involving perturbation of multiple Notch-mediated processes. Whereas statistical tests do show differences in severity of the phenotype between conditions, we do not feel these comparisons led to particular conclusions.**

6. Some methods are not very well documented, reducing reproducibility (e.g. TUNEL).

**There is a section with a detailed explanation of the TUNEL method in the methods section under the heading "Fluorescence in situ hybridizations, immunostainings, and TUNEL assay". For other methods that did not deviate from a published method, the corresponding paper is referenced.**

7. The labeling of many figures (e.g. Fig. 2C, 2E key, 2G asterisks and P ranges, Supp. 2C) is incredibly small. This reader would be grateful for labeling that could be read on a printed page or screen without magnification.

**We have increased the font size in all figures whenever possible. We have also updated all the figure callouts.**

**Have all data underlying the figures and results presented in the manuscript been provided?**
Large-scale datasets should be made available via a public repository as described in the *PLOS Genetics* [data availability policy](http://c05y1x9s.r.us-east-2.awstrack.me/L0/http:%2F%2Fjournals.plos.org%2Fplosgenetics%2Fs%2Fdata-availability/2/010f0192018df76c-c0f93fd5-f174-4be9-b269-95ac079e527c-000000/FpVtPufBDZLekma3JbtmW0kUjmA=177), and numerical data that underlies graphs or summary statistics should be provided in spreadsheet form as supporting information.

Reviewer #1: **No:** The single cell sequencing data from this paper is on a public database but it is set to not be released until September 2026. This prohibits the public to access and evaluate the data.

**The public database is set to release the sequencing data on September 2026 or at the time of publication, whatever happens first.**

Reviewer #2: Yes

Reviewer #3: Yes
